# Predictive capacity of COVID-19-related risk beliefs on weight management behaviors on a commercial weight loss program and speed of COVID-19 vaccination uptake: prospective cohort study

**DOI:** 10.1186/s12889-022-14481-2

**Published:** 2022-12-13

**Authors:** Annabell Suh Ho, E. Siobhan Mitchell, Jihye Lee, Andrew Steptoe, Heather Behr, Christine N. May, Andreas Michaelides

**Affiliations:** 1 Department of Academic Research, Noom Inc., 450 W 33rd St., NY 10001 New York, USA; 2 Moody College of Communication, The University of Texas at Austin, Austin, TX USA; 3 Department of Behavioural Science and Health, University College London, London, UK; 4 Department of Integrative Health, Saybrook University, Pasadena, CA USA

**Keywords:** Obesity, Weight management, Digital health, Mobile health, COVID-19

## Abstract

**Background:**

Recent work has shown that obesity may be a risk factor for severe COVID-19. However, it is unclear to what extent individuals have heard or believe this risk factor information, and how these beliefs may predict their preventive behaviors (e.g., weight management behaviors or COVID-19 preventive behaviors). Previous work has primarily looked at overall risk likelihood perceptions (i.e., not about obesity as a risk factor) within general populations of varying weight and concentrated on COVID-19-related preventive behaviors. Therefore, this prospective cohort study explored whether beliefs about obesity as a risk factor and overall risk likelihood perceptions predicted weight management and COVID-19 preventive behaviors over the next 16 weeks in individuals with obesity or overweight.

**Methods:**

Participants were 393 individuals in the US who joined a commercial weight management program in January, 2021. We leveraged the mobile program’s automatic measurement of real-time engagement in weight management behaviors (e.g., steps taken), while surveys measured risk beliefs at baseline as well as when individuals received COVID-19 vaccination doses (asked monthly) over the next 16 weeks. Mixed effects models predicted engagement and weight loss each week for 16 weeks, while ordinal logistic regression models predicted the month that individuals got vaccinated against COVID-19.

**Results:**

We found that belief in obesity as a risk factor at baseline significantly predicted greater engagement (e.g., steps taken, foods logged) in program-measured weight management behaviors over the next 16 weeks in models adjusted for baseline BMI, age, gender, and local vaccination rates (minimally adjusted) and in models additionally adjusted for demographic factors. Belief in obesity as a risk factor at baseline also significantly predicted speed of COVID-19 vaccination uptake in minimally adjusted models but not when demographic factors were taken into account. Exposure to obesity risk factor information at baseline predicted greater engagement over 16 weeks in minimally adjusted models.

**Conclusions:**

The results highlight the potential utility of effective education to increase individuals’ belief in obesity risk factor information and ultimately promote engagement or faster vaccination. Future research should investigate to what extent the results generalize to other populations.

## Background

There are multiple links between the COVID-19 pandemic and obesity. First, stay at home orders have curtailed many individuals’ usual healthy behaviors [1]. Second, the stress associated with the pandemic may have affected eating behaviors and weight management [2]. Third, obesity is considered a risk factor for severe outcomes among people who have been infected with the virus. A review from the early phase of the pandemic concluded that individuals with obesity had 113% higher risk of hospitalization if they had COVID, with 74% increased risk of ICU admission and 48% increased risk of death [3]. Many studies have since demonstrated that obesity increases risk of severe outcomes, and a few meta-analyses and studies also suggest that obesity could be a risk factor for increased susceptibility to COVID-19 (i.e., likelihood to test positive) [4–6].

There is growing public dialogue about obesity serving as a risk factor for COVID, with media articles describing increased risk of severe outcomes, as well as potential immunity and susceptibility implications, for those with obesity [7, 8]. This raises a pertinent question: for individuals with excess weight, how do their beliefs about obesity as a COVID-19 risk factor relate to future preventive behaviors (such as engagement with weight management behaviors, weight loss, and speed of COVID-19 vaccination uptake)? Research in risk communication indicates that individuals’ trust in risk information or information sources conveying risk is more important than exposure to risk information itself, and that individuals don’t always fully accept risk factor information they hear [9–11]. This suggests that individuals’ *belief* in obesity risk factor information may predict their preventive behaviors above *exposure* to this information.

Recent research has also shown that estimates of overall personal risk for COVID-19, not related to obesity, are associated with vaccination intentions and COVID-19 protective behaviors such as hand washing, mask wearing, and social distancing, particularly in the US. Specifically, as perceptions of likelihood are a key factor predicting preventive behaviors, a number of studies have examined the relationship between perceptions of likelihood (i.e., how likely am I to contract COVID-19?) and COVID-related protective behaviors or attitudes [12–17]. However, to date, most research has focused on general populations, not a population with obesity, as well as COVID-19-related behaviors (e.g., hand washing) and not weight management-related ones. Moreover, most research has focused on individual estimates of overall risk likelihood for COVID-19 and not group-related estimates for those who are in a high-risk group (e.g., those with obesity).

Therefore, in this study, we examined the predictive capacity of beliefs about obesity as a COVID risk factor and overall COVID risk likelihood on preventive behaviors (in terms of adherence to a weight management program and the speed of COVID-19 vaccination uptake) in a population of individuals who chose to join a publicly available weight management program during the pandemic. The specific preventive behaviors examined were engagement in program-measured weight management behaviors and weight loss given their relevance for this population as risk reduction behaviors, as well as speed of vaccination uptake, since risk likelihood perceptions are predictive of vaccination intent in previous literature. Given past research on risk likelihood beliefs and preventive behaviors suggesting that greater individual risk likelihood is associated with increased preventive behaviors, we hypothesized that 1) exposure to information about obesity as a risk factor, 2) belief about obesity as a risk factor, 3) personal risk likelihood perceptions, and 4) group (obesity) risk likelihood perceptions at program start would predict greater engagement and weight loss over 16 weeks, as well as faster speed of COVID-19 vaccination uptake.

## Methods

### Design

**Fig. 1:**
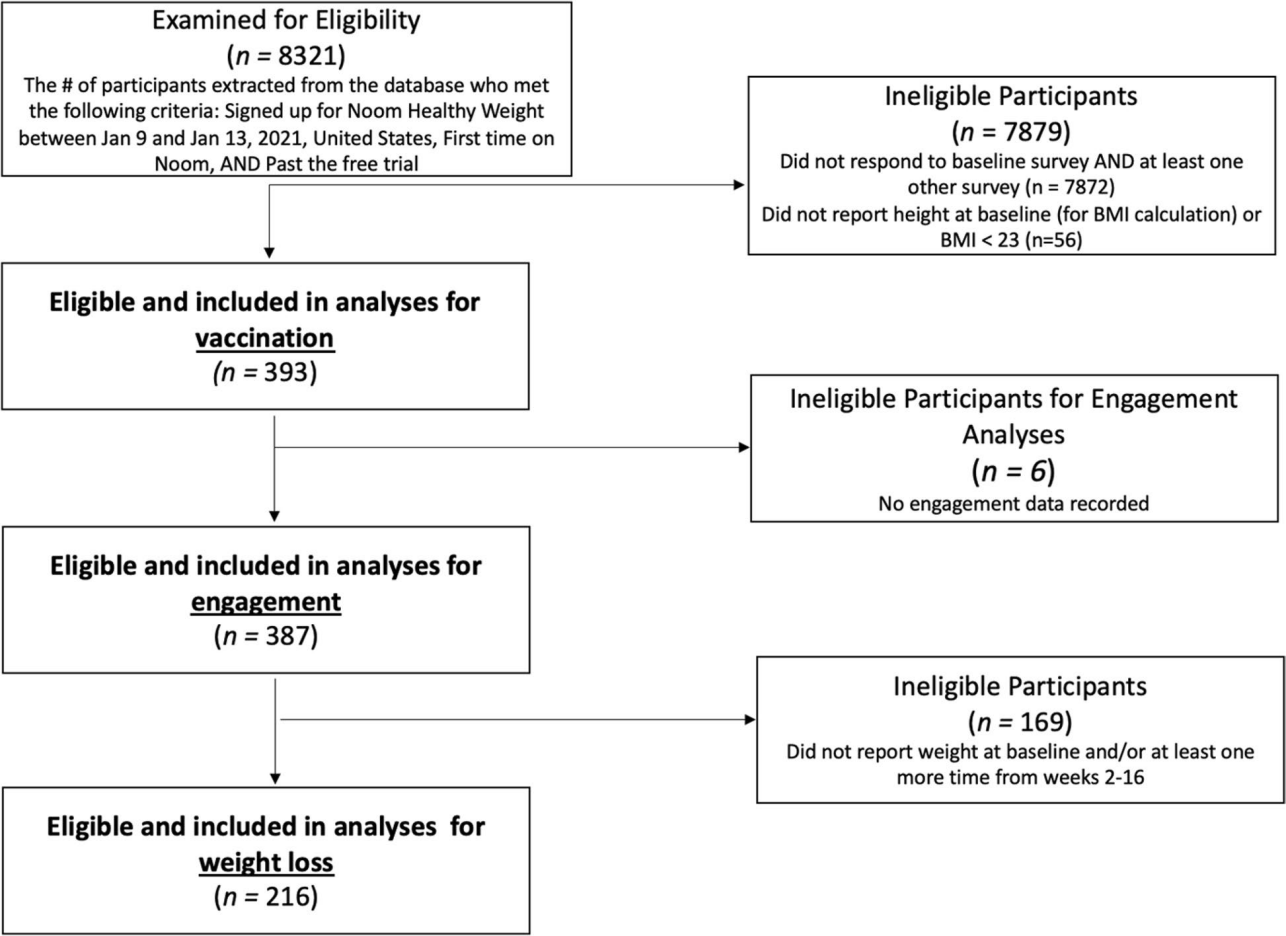
Diagram of study inclusion

This was a prospective cohort study approved by the Advarra IRB. Individuals who signed up for Noom (whether first-time or repeat), a commercial weight loss program, in January 2021, were from the US, and had at least a BMI of 23 were eligible and invited via email to participate in the 16 week study. The study took place remotely. Baseline surveys asked about risk beliefs. Surveys at 8 weeks and 16 weeks asked about vaccination status and the date(s) of vaccination doses. 393 individuals provided informed consent, with the knowledge that data would be used for research purposes, and completed the baseline survey and at least one other survey and were included in vaccination analyses. The sample sizes for engagement and weight loss analyses were based on available data. Participants with missing essential data (i.e., no record of engagement or did not report weight at baseline and at least once more) were excluded, leaving 387 participants in in engagement analyses and 216 in weight loss analyses (Fig. 1). All data were de-identified before analysis. All of these procedures were approved by the Advarra IRB.

Noom Weight is a commercial behavior change weight management program which is based on cognitive behavioral therapy (CBT), motivational interviewing, and third-wave CBT techniques, as well as behavior change techniques such as self-monitoring. Randomized controlled trials and observational studies have shown that Noom Weight can result in clinically significant weight loss [14, 15]. Individuals who sign up for the program receive access to the curriculum, in-app self-monitoring tools, individualized coaching via text message, and online groups.

### Patient and public involvement

No involvement.

### Measures

Exposure to information about obesity as a risk factor: “Have you heard any information (from the news, social media, or people you know) about increased risks of COVID-19 for individuals with obesity?” with responses ranging from “heard a great deal” (5) to “did not hear anything” (1), adapted from [18] and [19].

Belief about obesity as a risk factor: “How much do you personally believe there are increased risks of COVID-19 for those with obesity?” with responses ranging from “strongly agree” (7) to “strongly disagree” (1), written based on the exposure to information question.

Personal risk likelihood perception: “How likely do you think it is for you to get sick from COVID-19?” with responses ranging from “very likely” (5) to “very unlikely” (1), as utilized previously [20].^1^

Group risk likelihood perception: “How likely do you think it is for people with obesity to get sick from COVID-19?” with responses ranging from “very likely” (5) to “very unlikely” (1), adapted from the personal risk perception question.

Program engagement: Engagement was measured as the summed and normalized frequency of steps, weigh-ins, articles read, messages sent to the coach, exercises logged, foods logged, and app opens each week. These were automatically recorded in real time by the program and extracted from the program database. Weight, exercises logged, and foods logged were based on real-time user self-report (i.e., logs), whereas steps, articles read, coach messages, and app opens were automatically recorded regardless of self-report.

Weight loss: Participants self-reported weight on the program. Weight loss each week was calculated as each week’s reported weight subtracted from baseline weight; therefore negative values indicated weight loss and positive values indicated weight gain.

Speed of vaccination uptake: Participants reported the date that they had received each dose of a COVID-19 vaccine at baseline, 8 weeks, and 16 weeks. They also reported their intention to receive a vaccine dose. Booster doses were not available at the time of this study. We calculated an ordinal variable for the month of participants’ first vaccination dose, with the highest value indicating vaccination after July, 2021 or no intention to get vaccinated. Vaccines were available to the following populations during the time points in the study, with some individual variation among states in terms of specific requirements and timelines [21]: December 2020 (month prior to data collection): frontline essential workers, individuals 75 years of age and above; January 2021: individuals 65 and older and other essential workers; March 2021: individuals 16–64 with underlying medical conditions, including a BMI ≥ 30 kg/m2, and other essential workers; April 2021: all individuals age 16 and older.

Covariates: Demographics included age, gender, baseline BMI, education, race, ethnicity, and income. We also included local COVID-19 vaccination rates to account for any variability in vaccination distribution or possibilities of engagement outside of the home (e.g., going to the gym). Local COVID-19 vaccination rates were calculated using national average state level vaccination data from the CDC as of the study midpoint. Three categories were created: states with vaccination rate similar to national average (within M ± SD), states with vaccination rates lower than M (i.e., national average)—SD, and states with vaccination rate higher than M + SD.

### Statistical analysis

Ordinal logistic regressions were used to analyze speed of vaccination uptake. Mixed effects models, with random effects for each participant nested within time, and fixed effects of each risk belief plus covariates, were conducted to predict engagement and weight loss per week. Univariate models were conducted with one risk belief per model, and if more than one risk belief was significant (at the 0.05 level), a multivariate model was conducted. Variance inflation factors for multivariate analyses were all less than 2.5. We tested two models for each outcome: a minimally adjusted model taking age, gender, local COVID-19 vaccination rates, and baseline BMI into account, and a fully adjusted model including these variables plus education, race, ethnicity, and income. All weight loss models also adjusted for program engagement, which is a predictor of weight loss [22].

## Results

### Participant characteristics

The study sample was predominantly female, White, well-educated, and middle-aged (Table 1). The mean initial BMI was 32.7 (SD = 10.0) and average weight loss by 16 weeks was 6% or 6 kg (SD = 4% or 4.5 kg). Most of the sample (83%) reported receiving at least one vaccination dose by July (Table 2). At baseline, 66.7% reported feeling at least slightly motivated to join the program to lose weight because of COVID-19.

**Table 1:** Participant characteristics and engagement at baseline

Characteristic	Mean (SD) or N (%)
Initial BMI	32.74 (9.98)
Age	48.99 (14.11)
Gender	
Female	334 (85)
Male	57 (15)
Education	
Graduate degree	137 (35)
Undergraduate degree	115 (29)
Two-year degree or less	141 (36)
Race	
White	342 (87)
African American, Asian, and Other Races	35 (13)
Ethnicity	
Hispanic or Latino	12 (5)
Not Hispanic or Latino	375 (95)
Income per household	
Under $60,000	55 (14)
$60–100,000	101 (26)
$100,000 and over	161 (41)
Average weight loss by 16 weeks in kg	5.59 (SD = 4.49) kg
Average weight loss by 16 weeks in %	6.05% (SD = 4.29)
Average foods logged per week	48.17 (33.87)
Average exercises logged per week	3.24 (4.35)
Average weigh-ins per week	6.51 (1.65)
Average articles read per week	17.83 (12.75)
Average messages sent to coach per week	1.38 (1.75)
Average app opens per week	6.21 (1.71)
Motivation to lose weight on Noom because of COVID-19	
A great deal	35 (9)
A lot	57 (14)
Moderately	88 (22)
Slightly	81 (21)
Not at all	132 (33)

**Table 2:** Date of vaccination doses

Time of vaccination	Study Timeline	N (%)
Dose of vaccine at baseline	Baseline	47 (12%)
Dose of vaccine between Feb-March	2 months	203 (51%)
Dose of vaccine between April–May	4 months	72 (18%)
Dose of vaccine after 4 months or later (incl. never)	After 4 months	41 (11%)
Vaccine status unknown/missing	N/A	30 (8%)

Vaccination uptake speed is displayed grouped by study timepoint (e.g., 2 months, 4 months). Analyses used monthly data

### Minimally adjusted results

  Minimally adjusted univariate models (Table 3) showed that exposure to information about obesity as a risk factor predicted more engagement and marginally predicted faster vaccination uptake, but not weight loss (B = 0.29, t(1479) = 2.41, *p* = 0.02; OR = 0.87, 95% CI 0.75–1.01, *p* = 0.06; B = 0.06, t(815.80) = 0.65, *p* = 0.51). Beyond exposure, belief about obesity as a risk factor significantly predicted more engagement and faster vaccination uptake (B = 0.37, t(1478.90) = 2.83, *p* < 0.004; OR = 0.85, 95% CI 0.72–0.99, *p* = 0.04). It also predicted worse weight loss (B = 0.21, t(816.4) = 2.11, *p* = 0.03). Personal risk likelihood perceptions only predicted less engagement (B = -0.42, t(1478.60) = -2.68, *p* = 0.01), and group risk likelihood perceptions did not predict any of the outcomes.

**Table 3:** Results of univariate models predicting engagement, weight loss, and vaccination uptake speed

	**Engagement**		**Weight loss**		**Vaccination uptake speed**	
**Risk belief**	*Minimally Adjusted:* Coefficient (s.e.), *p*	*Adjusted:* Coefficient (s.e.), *p*	*Minimally Adjusted:* Coefficient (s.e.),* p*	*Adjusted:* Coefficient (s.e.), *p*	*Minimally Adjusted:* Odds Ratio (95% CI), *p*	*Adjusted:* Odds Ratio (95% CI), *p*
Exposure to obesity risk factor information	**0.29 (0.12), *p* = .02**	0.21 (0.15), *p* = .15	0.06 (0.09), *p* = .51	-0.02 (0.11), *p* = .88	*0.87 (0.75–1.01)*, *p* = *.06*	0.89 (0.75–1.07), *p* = .22
Personal belief in obesity as a risk factor	**0.37 (0.13), *p* = .004**	**0.51 (0.15), *p* < .001**	**0.21 (0.10), *p* = .03**	0.20 (0.13), *p* = .12	**0.85 (0.72–0.99), *p* = .04**	*0.85 (0.70–1.02), p* = *.09*
Personal risk likelihood perception	**-0.42 (0.16), *p* = .01**	*-0.33 (0.18), p* = *.07*	-0.03 (0.11), *p* = .81	-0.04 (0.13), *p* = .71	0.95 (0.78–1.15), *p* = .58	0.93 (0.75–1.15), *p* = .51
Group risk likelihood perception	-0.13 (0.19), *p* = .48	-0.17 (0.22), *p* = .43	0.10 (0.14), *p* = .45	0.15 (0.18), *p* = .38	0.86 (0.68–1.07), *p* = .18	0.96 (.74–1.25), *p* = .77

Univariate test statistics are displayed. Odds ratios are displayed for vaccination uptake speed, with coefficients displayed for engagement and weight loss

italic *p* < .10

bold *p* < .05

### Fully adjusted results

Adjusted models (Table 3) showed that exposure to obesity risk factor information no longer predicted engagement and vaccination uptake speed (B = 0.21, t(1149) = 1.45, *p* = 0.15; OR = 0.89, 95% CI 0.75–1.07, *p* = 0.22), and still did not predict weight loss (B = -0.02, t(633.4) = -0.14, p = 0.85) after sociodemographic factors had been taken into account. Belief about obesity as a risk factor still predicted more engagement and marginally predicted faster vaccination after controlling for demographic variables (B = 0.51, t(1148.8) = 3.27, *p* < 0.001; OR = 0.85, 95% CI 0.70–1.02, *p* = 0.09). It no longer predicted weight loss (B = 0.02, t(633.4) = 1.56, *p* = 0.12). Personal risk likelihood perceptions now marginally predicted worse engagement (B = -0.33, df(1148.6) = -1.83, p = 0.07), and group risk likelihood perceptions still did not predict any of the outcomes.

An adjusted multivariate model predicting engagement was conducted because exposure and belief about obesity as a risk factor were significant in univariate models. In this model predicting engagement using all demographics, exposure, and belief in obesity as a risk factor, only belief predicted significantly greater engagement (t(1147.8) = 3.08, *p* = 0.002).

### Covariates

In the adjusted models, older age was associated with higher engagement, and lower than average local vaccination rates were associated with less engagement (all *p*s < 0.002). Significant covariates for weight loss were gender and engagement, with greater weight loss among males and those with more engagement (all *p*s < 0.001). For vaccination speed, higher age and higher income were associated with faster vaccination uptake, with lower educational attainment associated with slower vaccination uptake (all *p*s < 0.02). Lower educational attainment was associated with slower vaccination uptake (all *ps* < . 009).

## Discussion

To our knowledge, this is the first study to assess whether beliefs about obesity as a COVID-19 risk factor and overall COVID-19 risk likelihood perceptions are predictive of preventive behaviors in a population motivated to manage their weight. The relationships between these risk beliefs and behaviors are complicated, but our results suggest that the extent to which individuals believe that obesity is a risk factor for COVID-19 may be a factor to consider and measure in a population with obesity. These beliefs at baseline predicted engagement in weight management-related behaviors over the next 16 weeks in both adjusted and minimally adjusted models, and weakly predicted speed of COVID-19 vaccination uptake. These relationships were significant even in this population, which is likely highly motivated both to engage in weight management behaviors and to get vaccinated. In fact, most participants also reported they were at least partly motivated by COVID-19 to join this weight management program, and that most had received a vaccination dose by April 2021. This raises the question of whether the effects would be larger in less motivated populations that would show more variance in vaccination uptake speed or program engagement. Additionally, consistent with previous studies of the general US population [13, 15], we found that age, gender, income, and education predicted vaccination uptake speed in this population. Another novel finding was that low local vaccination rates predicted worse engagement. This could be because individuals found it challenging to exercise outside of the home in areas in which many were not vaccinated, whether due to site closures or fear of contracting COVID-19.

Belief in obesity as a risk factor predicted engagement more strongly than mere exposure to this information. Our results may indicate that while individuals hear information about obesity as a risk factor, what is more predictive is whether they personally believe this information. This echoes previous work about how individuals’ trust in risk information predicts behavioral outcomes [9, 10], and a study showing that individuals who were exposed to risk factor information did not incorporate it in their understanding of their risk [11]. Future research should explore how belief in obesity as a risk factor compares to other attitudes or beliefs that predict COVID-19-related preventive behaviors.

Surprisingly, overall risk likelihood perceptions about COVID-19 (i.e., not about obesity as a risk factor) did not strongly predict speed of vaccination uptake or engagement. While previous studies have shown that overall risk likelihood perceptions predict behaviors directly related to COVID-19 (e.g., social distancing) and vaccine hesitancy in general adult populations [13, 15–17, 23], our results align with studies finding non-significant associations in populations at high risk for COVID-19 (e.g., those with respiratory disease) [24–26]. In a population that already perceives itself to be at high risk, perhaps additional variation in overall perception of risk likelihood is not explanatory. With recent work suggesting a strong link between obesity and *severity* of COVID-19 outcomes (e.g., [27]), it should also be noted that we found similar results when analyzing a follow-up question asking about perceptions of personal severity (if you were to get sick, how severe would it be?). Just as for personal likelihood, there were no significant associations between personal severity perceptions and outcomes. The fact that there were significant associations for beliefs about obesity as a risk factor, but not for personal likelihood or severity, suggest that this construct operates differently than personal likelihood or severity perceptions. Our results suggest that beliefs related to *obesity as a risk factor*, more than overall COVID-19 risk, should be examined more in future research and public health efforts for a population with overweight or obesity motivated to manage their weight, especially as biological research increasingly explores obesity’s role as a COVID-19 risk factor (e.g., [28]. Future research should examine the specific components of this belief and how they operate to influence preventive behaviors. Another unexpected finding was that belief in obesity as a risk factor predicted better engagement but not weight loss. Significant covariates predicting weight loss (male gender and engagement) matched previous work [22, 29], which perhaps means those factors explain weight loss much more than these beliefs. Future studies should explore the relationships with weight loss more deeply.

This study had several limitations. First, as previously mentioned, when measuring risk beliefs, we focused on likelihood (i.e., perceived susceptibility to COVID-19) because of previous empirical and theoretical work showing that likelihood is a key factor driving preventive behavior [12–17]. Because of the many recent studies showing that obesity is linked to *severity* of COVID-19 outcomes, future work should include and examine severity and whether it differs at all from likelihood perceptions. Future work should also break down the obesity as risk factor question into risk factor for likelihood (i.e., susceptibility of catching COVID-19) and for severity (i.e., severe outcomes). Another limitation is that we did not measure other predictive factors for speed of vaccination uptake (e.g., attitudes towards vaccines) or preventive behaviors, such as health factors that could increase the salience of COVID-19 risk. For instance, conditions such as chronic respiratory disease or cardiovascular diseases, which are also risk factors for severe COVID-19 that can co-occur with obesity, as well as stress or anxiety related to COVID-19, could influence willingness to engage in preventive behaviors or receive vaccination [30, 31]; future studies should separate the individual effects of each of these factors from those measured in this study. Additionally, self-reported weight was used rather than objective measurement of weight.

Other limitations include the sample used in this study, which may not generalize to general populations with obesity who have not joined a commercial program to manage their weight. Also, the population was mostly White, female, and highly educated. This is fairly typical of individuals who currently sign up for the program, and we found similar demographic predictors of vaccination uptake speed, engagement, and weight loss compared to previous work [13, 22, 29, 32]. The sample was also recruited at a specific time point during the pandemic (in January 2021). This time point was chosen in order to track, in real time and prospectively, vaccination uptake, as this was the period in which vaccinations were being rolled out to the US population. However, this time period, almost a year into the pandemic, raises the possibility that the individuals who had been most motivated to engage in preventive behaviors had already signed up previously, which could result in stronger relationships than seen here. It should be noted that the majority of this sample was motivated to lose weight because of COVID-19; thus, it is not likely that the former sample would result in entirely *different* relationships, but instead potentially could show different magnitudes of the same relationships (e.g., stronger effects). Another important point about the time period is that data collection occurred shortly after New Years, which raises the possibility that this was a less motivated or successful sample for long-term weight loss for those focused on New Years resolutions [33]. However, average weight loss for this sample was similar to that of Noom samples who signed up during other parts of the year [34, 35]. Still, there could be other potential differences depending on the time period in which this data was collected, so future research should ascertain the extent to which the results of this study generalize to other populations and time periods. Another limitation is that the analyses were based on available data; given that there were marginally significant results, future studies should aim to replicate these findings ensuring that the sample size provides sufficient power to detect the effect sizes reported here. Finally, some participants (12%) had already been vaccinated by the time of the baseline survey, which potentially decreased the variation we could observe in vaccination uptake speed.

As the COVID-19 pandemic continues and infection rates remain or reach high levels in many countries, there is a need for effective obesity management. This exploratory study offers some public health implications. First, public health messaging may seek to educate individuals on obesity’s potential role as a COVID-19 risk factor and to focus on ways to encourage greater belief that this information is valid, in order to improve engagement in weight management behaviors such as exercise, and to a lesser extent, speed of vaccination uptake. The relationship between risk factor belief and vaccination uptake was small in adjusted models and should be interpreted with caution. Future research should investigate if there are any potential practical implications, as a one unit increase in belief was associated with 15% lower odds of a one month delay in vaccination uptake in this study even when adjusting for demographic variables. Potential strategies for increasing risk factor beliefs could include personalizing risk factor information or communicating it via personal narratives, rather than through objective facts [36, 37]. This should be done with care and consideration of possible weight bias and shame internalization. Another implication from our findings is that overall risk likelihood perceptions (i.e., likelihood of getting COVID) may not be particularly informative for predicting vaccination uptake speed or weight management behaviors in a population with obesity or overweight. Future studies should investigate how and when overall risk likelihood perceptions are useful to predict future behaviors in this population. Finally, our findings that local vaccination rates were associated with engagement suggest that individuals living in areas with lower-than-average vaccination rates should be provided more support for engagement in weight loss behaviors, particularly during surges in the pandemic that make it difficult to exercise outdoors or in public establishments. These possibilities should be tested by future research.

## Data Availability

The datasets generated and/or analyzed during the current study are available from Noom Inc. but restrictions apply to the availability of these data, which were used under license for the current study, and so are not publicly available. Data are however available from the authors upon reasonable request and with the permission of Noom Inc.

## Footnotes

1 A follow-up question asked, “**If** you were to get sick, how serious do you think it would be?” This showed the same pattern of results as the likelihood question.
